# New eHealth Users After COVID-19: Adoption and Resistance in Spain

**DOI:** 10.3390/healthcare14060807

**Published:** 2026-03-21

**Authors:** Irene Loureiro-Álvarez, Antón Lodeiro-Vázquez, Bran Barral-Buceta

**Affiliations:** Department of Political Science and Sociology, Faculty of Political and Social Science, University of Santiago de Compostela, Avda. Dr. Ángel Echeverri, S/N, 15782 Santiago de Compostela, Spain; irene.loureiro@rai.usc.es (I.L.-Á.); antonlodeiro.vazquez@usc.es (A.L.-V.)

**Keywords:** eHealth, digital health adoption, COVID-19, Spain, digital inclusion, health equity, ageing, mobile health

## Abstract

**Highlights:**

**What are the main findings?**

- Mobile applications have established themselves as the main channel for accessing digital health in Spain, driving most of the increase in eHealth use following the pandemic.
- Age stands out as the most decisive socio-demographic barrier to access: older people are significantly less likely to use eHealth, while the use of digital devices clearly improves access.

**What are the implications of the main findings?**

- Public policies must prioritise digital inclusion, especially strengthening digital health literacy among older people and groups with less technological familiarity.
- The transition to mobile-centred access requires designing digital services to ensure accessibility, usability and ease of use as eHealth becomes a standard component of healthcare.

**Abstract:**

**Background/Objectives:** The advent of the pandemic catalysed the global adoption of digital health services. In Spain, this transition has markedly influenced eHealth accessibility, particularly through mobile-based technologies. This study compares reported real-access experiences of users of digital health at two key moments—2018 (pre-pandemic) and 2025 (post-pandemic)—to identify how access determinants have evolved. **Methods:** The evolution of users who have accessed digital health resources and their characterization, including the specification of who uses websites and mobile applications, was analyzed using binary logistic regression models for each year. These models incorporated sociodemographic characteristics and patterns of device usage as predictors. **Results:** Access to digital health services increased significantly between 2018 and 2025. The proportion of the population using such services increased from 50.2% to 85.4%. The use of mobile applications grew from 24.4% to 80.2%. In 2018, access was linked to a wider range of factors, including age, education, municipality size, self-rated health, and computer or tablet use. In contrast, the 2025 model revealed a more concentrated set of determinants. Age emerged as the primary barrier, especially >65 years, reducing the likelihood of eHealth access. Meanwhile, frequent use of digital devices (e.g., computers, smartwatches) was positively correlated with accessing eHealth. **Conclusions:** The study reveals an increase in access to digital health services in Spain, accompanied by shifts in the factors influencing this access. Notwithstanding technological advances, the digital divide could persist as a major impediment to access for the groups mentioned earlier. This analysis suggests the need for targeted digital inclusion measures, especially as mobile platforms are becoming the main entry point to healthcare services.

## 1. Introduction

Health and healthcare provision have historically been one of the pillars on which the welfare state is built. In most European countries, the organisation of health services reflects not only a policy of care, but also a philosophical vision of the social contract [1]. From the Scandinavian models of universal coverage and public funding to mixed systems that combine state responsibility with private participation, healthcare is conceived as a right and an indicator of the degree of civic development [2]. Spain occupies an intermediate position: a quasi-universal system supported by general taxation, which guarantees basic care for all citizens, but in which complementary financing and the unequal territorial distribution of resources generate a mosaic of seventeen regional subsystems [3,4,5], with varying levels of digitalisation, interoperability and technological literacy among healthcare personnel [6,7,8]. At the same time, private provision has also gained ground in recent years [5,9].

The 1986 “General Health Law” consolidated the principle of “equal access for equal need”, establishing an institutional architecture geared towards equity and decentralisation [10]. During the first decade of the 21st century, technological advances opened the door to a progressive transformation of the healthcare model. eHealth, understood as the systematic incorporation of Information and Communication Technologies (ICT) into healthcare processes, began to redefine the boundaries of medical care. This concept encompasses more than the digitalization of services; it represents an institutional commitment to enhancing the efficiency and quality of care through global information networks [11]. Other authors have described this process as a “cultural transformation” in medicine, in which objective data and two-way communication are gradually replacing the traditional paternalistic model [12]

The field of eHealth provision has been impacted by significant developments in technology, with mHealth (mobile health) emerging as the most tangible expression of this change. The proliferation of smartphones and wearable devices has enabled a shift in the point of care from medical offices to the patient’s everyday life [3]. Mobile applications for monitoring physical activity, measuring vital signs, and managing medical appointments transformed the role of the user, who went from being a passive recipient to an active participant in the management of their health [13].

However, before 2020, the penetration of these tools was still limited and concentrated in young, urban and highly educated sectors. The typical patient of face-to-face services—over 65 years of age, living in rural or semi-urban areas and with a low level of education—was left out of this silent revolution [14]. eHealth was perceived as an innovative complement, not as a structural necessity of the system.

The COVID-19 pandemic may have significantly accelerated the ongoing digitalisation processes in the Spanish healthcare system, reshaping certain dynamics of access and use. Home confinement, mobility restrictions and hospital saturation caused a veritable “systemic shock” in the sense proposed by Sabatier [15,16,17]. The impossibility of maintaining face-to-face services forced the immediate migration of multiple services to the digital environment. Teleconsultation, electronic prescriptions, doctor-patient communication platforms, and infection tracking applications have become essential tools for sustaining healthcare. This phenomenon has been documented in recent studies conducted in Spain [18] and a comparative analysis with Portugal [19].

From a European perspective, according to data from the European Commission [20], Spain is among the fast-trackers, in a medium-high position with regard to the evolution of eHealth. In this regard, its strengths lie in the institutional provision of services and technological competence. However, the Spanish model has less presence and momentum in terms of citizen trust and participation in digital areas, compared to the leading states (trendsetters).

Despite the gradual improvement in the maturity of the system [20], Spain still faces limitations in terms of the structure of its healthcare system. The decentralisation of health competences, which also extends to eHealth, raises questions about speed and equity of access, guarantees that are still pending in the field of digital health.

This abrupt transition highlighted both the potential and the shortcomings of the system. The pandemic acted as a catalyst for innovation, but it also exposed structural inequalities in access and digital competence. For many older people or those with limited technological resources, the increase in digital channels during COVID-19 restrictions may have generated feelings of isolation or a perception of contact with the healthcare system [21,22]. For other groups, however, it was an opportunity for empowerment and self-management [10]. The paradigm shift brought with it the emergence of the ePatient, a concept coined by Ferguson and Frydman to describe an informed, connected individual capable of actively participating in decisions that affect their well-being, ultimately leading to what Lupton calls the digitised healthy citizen [23,24]. The doctor-patient relationship was transformed into a horizontal dialogue, where information no longer flows in a single direction. Healthcare professionals began to play a “guiding” role in a distributed knowledge ecosystem. However, this transition generated tensions: some professionals expressed fears about the dehumanisation of care or bureaucratic overload associated with the use of digital platforms [25].

During the pandemic, perceived usefulness increased significantly, driven by necessity: digital tools became the viable channel for accessing care [26,27,28,29]. This exceptional context meant that even people with low technological competence perceived tangible benefits from their use, temporarily increasing the adoption rate [26,27]. However, once the critical phase was over, gaps related to literacy and trust re-emerged [29].

Digital health literacy (eHealth literacy), defined by Norman and Skinner [30] as the combination of traditional, informational, scientific, media, and computational skills, proved to be a decisive factor. It is not just a matter of knowing how to use an application, but of being able to transform information into meaningful decisions, following Sen’s postulate of basic capabilities [31,32]. Subsequent studies show that the pandemic acted as an accelerator of learning: Großschädl et al. [33] found that adults over 50 reported higher literacy levels than young people, interpreting the crisis as a forced learning process. Even so, the digital divide persists, with citizens in rural areas or with lower levels of education continuing to encounter barriers to access and understanding [34]. Healthcare is no stranger to this situation either [35,36,37].

In this context, the welfare state is being redefined. It is no longer enough to guarantee universal coverage; it is necessary to ensure effective digital access to public services. Health equity in the 21st century depends as much on the right to care as on the right to connection. In this sense, the pandemic served as a laboratory of institutional stress: it forced governments to rethink their digital infrastructures, staff training, and information governance models [10].

The consolidation of the post-pandemic digital health ecosystem has revealed a paradox: while technology expands the possibilities for access and personalisation of care, it also highlights new structural inequalities. The notion of “digital efficiency” cannot be understood in isolation from social justice. As Norris [38] warns, digitalisation does not eliminate gaps, but rather reconfigures them, shifting material inequalities into the realm of knowledge and technological competence.

The digital divide in healthcare is shaped not only by access to technology but also by sociodemographic, cultural, and psychological factors. Previous studies show that age, education, gender, and geographical context influence attitudes toward healthcare technologies [39,40,41]. Younger individuals tend to adopt mobile health applications and telemedicine more rapidly, whereas older adults are often more cautious, partly due to concerns about reduced human interaction [42]. However, positive attitudes may emerge when digital tools are perceived as enhancing independence and security among older users [40]. Educational level is another key determinant: individuals with higher education tend to have more favourable attitudes toward eHealth and greater confidence in its reliability, while those with lower education face difficulties in understanding digital medical information [41,43]. Gender also introduces significant differences, with several studies indicating that women tend to underestimate their own technological literacy and express greater concern about ethical or privacy risks associated with health technologies [44,45,46].

The cultural change resulting from the pandemic extends beyond digital health. Healthcare digitalisation has become intertwined with the expansion of emerging technologies, including Artificial Intelligence (AI), which represents a new frontier in the post-pandemic ecosystem. Far from constituting a break with the past, AI can be interpreted as a natural extension of the evolution that began with eHealth, amplifying existing trends: it automates the analysis of medical images, optimises resource allocation and facilitates more accurate diagnoses [47,48,49,50,51]. However, its implementation also reopens ethical debates about algorithmic transparency, data privacy, and the risk of dehumanisation of care [52]. These concerns reflect a constant tension between efficiency and equity: technology promises universality, but it can deepen inequalities if it is not accompanied by digital inclusion policies.

The present study examines the factors determining access to digital health (eHealth) in Spain, and the ways in which these factors have evolved in a context of rapid digital transformation. By comparing scenarios from before and after the pandemic, the research analyses both the expansion of access and the changing role of sociodemographic factors over time. In contradistinction to extant surveys administered by official sources, such as the Centro de Investigaciones Sociológicas, this study provides a more detailed analysis of eHealth and enables a systematic longitudinal comparison.

In this context, the article focuses on two key moments: 2018, prior to the COVID-19 pandemic, and 2025, following the expansion of the digital health ecosystem. The research question guiding this work is: how has access to eHealth changed during this period?

In order to address this question, the study pursues two main objectives: firstly, to quantify the expansion of access to eHealth by comparing pre- and post-pandemic scenarios; and secondly, to identify the sociodemographic factors associated with such access, as well as how their explanatory role has evolved over time.

The analysis is guided by the hypothesis that, despite the overall expansion of digital health access, age remains a significant barrier, while the widespread adoption of smartphones contributes to broadening access to eHealth services.

## 2. Materials and Methods

### 2.1. Study Design

This study follows a quantitative research design based on the analysis of two cross-sectional surveys specifically designed and implemented by the research team. The objective of this approach is to examine the relationship between Spanish citizens and access to eHealth services, as well as to compare patterns before and after the COVID-19 pandemic.

The first survey, conducted in 2018—prior to the COVID-19 pandemic—is entitled “Use and Attitudes towards eHealth in Spain” (hereinafter, the 2018 Survey). The second survey, conducted in 2025, is entitled “eSactiv: Ageing and eHealth in Spain” (hereinafter, the 2025 Survey).

Prior to their utilisation in the fieldwork, the instruments underwent a series of rigorous review and validation processes. These processes comprised triangulation of research team members and consultations with experts in the field of digital health. These processes were conducted in accordance with stringent bioethical committee protocols, which were meticulously adhered to prior to the commencement of fieldwork.

In order to ensure conceptual comparability, several questions were adapted from commonly used indicators in national surveys on ICT use and digital practices in Spain (CIS and INE) and Europe (Eurostat). The questionnaire was designed to allow consistent measurement across the 2018 and 2025 survey waves.

#### 2.1.1. Study Setting, Participants and Inclusion Criteria

The 2018 survey was conducted across Spain, covering all 17 autonomous communities (CC. AA.), with the exception of the autonomous cities of Ceuta and Melilla. The 2025 survey also targeted the Spanish population nationwide, with a specific sub-sample of individuals aged 65 and older. The analysis focused on identifying sociodemographic and technological factors associated with eHealth access.

The target population for both surveys was adults residing in Spain. The inclusion criteria were being 18 years of age or older and residing in Spain. These criteria defined the study universe and ensured that the samples were representative of the adult population residing in Spain.

#### 2.1.2. Sampling Method and Sample Size Justification

The surveys employed a probabilistic sampling strategy designed to ensure representativeness of the Spanish population. A random sampling method was applied, with stratification based on key sociodemographic variables to ensure adequate territorial and demographic coverage.

The 2018 survey included a total sample of 1695 respondents. Representativeness across the entire national territory was ensured through selection criteria based on gender, age and habitat, covering the 17 autonomous communities (CC. AA.), excluding the autonomous cities of Ceuta and Melilla. The survey achieved a confidence level of 95% with a margin of error of ±2.45% for the overall sample.

The 2025 survey initially had 1599 respondents, with a representative subsample of people aged 65 and over (N = 553). To ensure national representativeness, weighting procedures were applied according to autonomous community (excluding Ceuta and Melilla), gender and age group. After weighting adjustments, the effective sample size used in the analysis was 1086 observations. The survey achieved a 95% confidence level with a margin of error of ±2.7%.

#### 2.1.3. Data Collection Procedures

In the 2018 Survey, data were collected through computer-assisted telephone interviews (CATI). The 2025 Survey implemented a mixed-mode data collection design. Individuals aged 18–64 years completed computer-assisted web interviews (CAWI), whereas participants aged 65 years and older were surveyed using CATI. This methodological decision was based on logistical considerations and supported by prior evidence on internet and technology use among adults under 65. Fieldwork for the 2018 Survey was conducted between 24 May and 21 June and the 2025 survey was conducted between 30 May and 8 July 2025.

Interviewers received prior training to ensure standardized administration of the CATI questionnaires. In the CAWI modality, participants accessed the survey through a secure online platform. Informed consent was obtained from all participants prior to participation, and anonymity was guaranteed throughout the process. In both surveys a pilot test was conducted to assess clarity, length, and internal consistency of the questionnaire before full deployment.

### 2.2. Variables

Both surveys allow for the comparison of two contrasting scenarios (pre- and post-pandemic) and the quantification of the evolution of access to digital health. The analysis focuses on identifying the sociodemographic and technological factors associated with such access. To this end, two binary logistic regression models were developed—one for each survey—capable of establishing correlations between the factors that estimate greater use of eHealth tools, as well as establishing comparisons between this access before and after the COVID-19 pandemic. This technique was chosen due to the dichotomous nature of the dependent variable, the availability of data, and the interpretation of the subsequent results. For the selection of sociodemographic variables (see Table 1), it should be noted that those present in both surveys were collected, illustrating a fair comparison between the final models. Thus, the following stratification variables were chosen: gender, age, household composition, place of residence, educational level, income, employment status, chronic disease, and self-perceived health status.

Regarding the variables relating to the use of technological devices, these focus on the use of mobile phones, tablets, computers (both laptops and desktops) and smart watches. It is important to note that certain devices were not as prevalent at the two specified points in time, which also suggests the possibility of technological advancements and trends in wearable devices. Nevertheless, although certain response categories emerge, comparability is maintained and technological updates are also taken into account, thereby enabling the assessment of the impact of each tool.

Finally, a new variable, Access to eHealth, has been created, resulting from the merger of questions on access to the digital health system via both websites and mobile applications. This is the result of combining, in each of the surveys, questions about access to the digital healthcare system via the website and/or mobile applications.

It should be noted that these variables have undergone an additional recoding process, being configured as dummy variables —where 0 represents the absence of a condition, while 1 represents the presence of a phenomenon— in order to adapt them to binary logistic regression models.

### 2.3. Statistical Analysis

Following the methodological proposal, this section describes certain issues related to the statistical analysis proposed for this research. The analyses were performed using IBM SPSS Statistics (Version 29.0.2.0 (20)) and RStudio statistical software (Version 2026.01.1).

First, the results regarding access to digital health services, whether via the web, mobile devices, or both, are presented descriptively in order to illustrate the evolution in recent years, as well as the impact of COVID-19 on the spread of these tools.

Regarding the binary logistic regression analysis, a parsimonious model was constructed that included only those variables that were statistically significant in explaining access to eHealth services. An analysis process was carried out in which the variables were systematically evaluated and sequentially retained or excluded based on their contribution to the model fit, allowing the final model to focus on the most relevant predictors.

Two models were created, one per survey, the results of which are distributed across three graphical elements. The first, corresponding to the “Model Summary”, contains the main statistical elements of this analysis, offering an overview of each of the survey results. Therefore, different pseudo R2 values are presented—McFadden’s R2, Cox and Snell’s R2, Nagelkerke’s R2 and Tjur’s R2—which are key measures for evaluating the degree of fit of the model to the data. Following this section, the table of “Model Coefficients” is presented, which indicates the strength of the independent variables on the dependent variable. In this regard, different statistics are included, such as the Beta Estimator (B), the Standard Error (SE), the Z value, the significance level (*p*), and the different Odds Ratios (OR). As a complement, these tables are accompanied by a graphical representation of the ORs with their corresponding confidence levels, using forest plots.

Finally, the appendices section is completed with additional information intended to evaluate the overall significance and internal consistency of the estimated models. Specifically, it includes the Omnibus likelihood ratio test, used to test the joint significance of the model. Likewise, the Variance Inflation Factor (VIF) is reported as a diagnostic statistic of multicollinearity among the independent variables (see Appendix A).

### 2.4. Ethical Considerations

The study is based on two survey waves conducted in 2018 and 2025. In both cases, participants were contacted by trained fieldwork staff using non-personal telephone and email databases. Prior to participation, respondents were informed about the purpose and funding of the study, the identity of the research team and responsible institutions, and the intended use of their anonymised data. Participation was voluntary, and informed consent was obtained verbally from all participants before proceeding with the questionnaire. The study was conducted in accordance with the principles of the Declaration of Helsinki.

The first survey was conducted in 2018, prior to the establishment of the institutional Bioethics Committee at the University of Santiago de Compostela. Nevertheless, the study complied with the applicable national and European data protection regulations in force at the time, including Regulation (EU) 2016/679 General Data Protection Regulation (GDPR) and its Spanish complement, Organic Law 3/2018 on the Protection of Personal Data and Guarantee of Digital Rights (LOPDGDD). The survey was anonymous, non-interventional, and based on voluntary participation with prior information provided to respondents.

The second survey, conducted in 2025, received ethical approval from the Bioethics Committee of the University of Santiago de Compostela (reference USC 44/2024, approved on 23 May 2024). All procedures complied with relevant international ethical standards for research involving human participants.

No personally identifiable information was collected. All data were processed anonymously, stored securely, and used exclusively for academic research purposes.

## 3. Results

The results are presented in four sections: (1) a description of the sociodemographic characteristics of the sample; (2) an overview of the expansion of access to digital health between 2018 and 2025; (3) factors associated with access in 2018; (4) factors associated with access in 2025; and (5) a comparison between the two models.

### 3.1. Sociodemographic Characteristics of the Sample

Table 2 presents the sample distribution by gender and age group, for both rounds of the survey. This distribution reflects the sociodemographic structure of the Spanish population, thus ensuring the representativeness of the samples.

The final sample size was N = 1695 participants for the 2018 survey and N = 1086 for the 2025 survey, after applying the weighting procedures.

### 3.2. Access to Digital Health in 2018 and 2025: Overview

Table 3 shows a comparison of access to digital health services in 2018 and 2025, specifically evaluating two main modalities provided by the regional health system: access to websites and access to mobile applications.

In terms of access to health websites, 50.2% of respondents had accessed them at some point in 2018, while in 2025 this percentage of access rises moderately to 59%.

In the case of access to mobile health applications, the projected changes are very significant. In 2018, only 24.4% of participants used mobile applications to access health services, compared to 75.6% who had not accessed them. In contrast, in 2025, access increases significantly, reaching 80.2% of the population who do access them.

If access via at least one of the two channels (website and/or mobile application) is considered, a substantial increase can be observed between 2018 and 2025. In aggregate terms, access to digital health has gone from covering approximately half of the population (50.2%) in 2018 to reaching a large majority (85.4%) in 2025.

### 3.3. Access in 2018

Table 4 shows the results of model 1 estimated to explain access to digital health in Spain in 2018 via the website and/or mobile applications. The results show that the model has moderate and statistically significant explanatory power (x^2^ = 125; *p* < 0.001). The pseudo-R^2^goodness-of-fit indicators are 0.086 (McFadden), 0.112 (CoxSnell) and 0.150 (Nagelkerke). Furthermore, as presented in Appendix A, all values estimated using Variance Inflation Factors (VIF) remain well below conventional cutoff thresholds, confirming the absence of multicollinearity among the predictors.

Looking first at the estimated coefficients (B), a set of variables is identified that have negative effects on the probability of access to digital health in 2018. Specifically, belonging to the over-65 age group is associated with a negative and statistically significant coefficient (B = −0.631; *p* = 0.014). Similarly, having secondary education shows a significant negative effect at 99% confidence (B = −0.759; *p* < 0.001), as does reporting regular health status (B = −0.605; *p* = 0.003).

Conversely, several variables have positive coefficients, suggesting a higher probability of access to digital health. Residing in municipalities with between 25,000 and 100,000 inhabitants is positively associated with access (B = 0.403; *p* = 0.009), as is suffering from a chronic disease (B = 0.577; *p* < 0.001). Likewise, variables linked to the use of digital technologies show significant positive effects: tablet use has a positive and significant coefficient (B = 0.394; *p* = 0.003), while computer use has the highest coefficient in the model (B = 1.216; *p* < 0.001).

**Figure 1:**
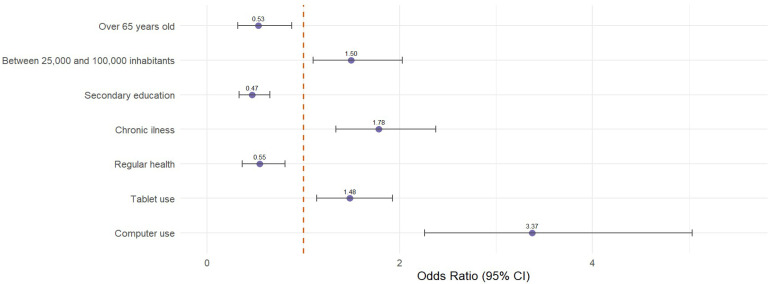
Forest Plot Model 1.

Figure 1 graphically summarises these results using odds ratios (OR) and their 95% confidence intervals, taking the dashed vertical line at OR = 1 as a reference. Predictors whose intervals do not intersect this line show statistically significant associations with access to digital health in 2018.

In the range below unity are the variables associated with a lower probability of access: being over 65 years of age (OR = 0.532), having secondary education (OR = 0.468) and reporting regular health status (OR = 0.546). Conversely, the range above OR = 1 contains factors that increase the probability of access, including living in municipalities with between 25,000 and 100,000 inhabitants (OR = 1.496), having a chronic disease (OR = 1.781), the use of a tablet (OR = 1.482) and, most notably, the use of a computer, which more than triples the probability of access to digital health in 2018 (OR = 3.374).

### 3.4. Access in 2025

Table 5 shows the results of model 2 estimated for access to eHealth in 2025 via the website and/or mobile applications. The overall contrast of the model is statistically significant (x^2^ = 180; *p* < 0.001), indicating an adequate joint fit of the variables included. The pseudo-R^2^goodness-of-fit indicators reach values of 0.323 (McFadden), 0.163 (CoxSnell) and 0.385 (Nagelkerke), reflecting a notable increase in the explanatory power of the model. VIF diagnostics indicate no evidence of multicollinearity among the independent variables (see Appendix A), thereby supporting the stability and reliability of the estimated coefficients.

Looking first at the estimated coefficients (B), there is a large negative effect associated with the group of people over 65 years of age (B = −2.185; *p* < 0.001), indicating a significantly lower probability of access to eHealth in 2025.

Conversely, several positive coefficients are identified. The 35–49 age group has a positive and statistically significant effect (B = 1.539; *p* = 0.024). This suggests that individuals in this age range have a higher likelihood of using eHealth services in 2025. Likewise, computer use maintains a significant positive effect (B = 1.015; *p* < 0.001).

Finally, a variable linked to the use of emerging digital devices is incorporated, whose effect is also positive and significant. Specifically, the use of smartwatches is associated with a higher probability of access to eHealth (B = 0.678; *p* = 0.027).

**Figure 2:**
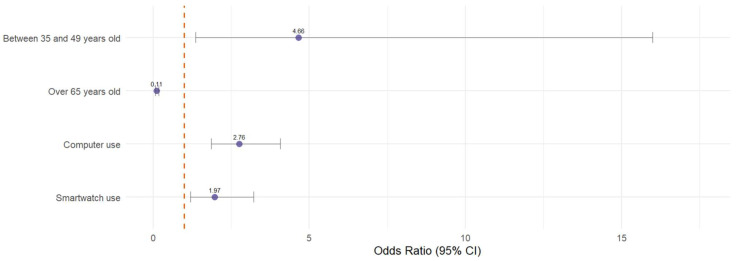
Forest Plot Model 2.

Figure 2 provides a concise visual summary of the results of Model 2 by displaying the odds ratios (OR) and their 95% confidence intervals, using the dashed vertical line at OR = 1 as a reference point.

The variable corresponding to being over 65 years of age (OR = 0.112) is the only one in the range below unity, indicating a substantially lower probability of accessing eHealth in this age group. Conversely, the range above OR = 1 concentrates the factors associated with a higher probability of access, notably the age group between 35 and 49 years old, which has the highest odds ratio in the model (OR = 4.661), followed by computer use (OR = 2.760) and smartwatch use (OR = 1.970).

### 3.5. Comparison of Models

Finally, Table 6 presents a summary comparison of the estimated coefficients (B) and odds ratios (OR) of the models analysed. The comparison between the models estimated for 2018 and 2025 allows us to identify relevant changes in the determinants of access to eHealth in Spain, both in terms of explanatory power and in the intensity and configuration of the effects associated with the variables included.

Firstly, there is a notable increase in the explanatory power of the model in 2025 compared to 2018, as reflected in the pseudo-R^2^ values. While in 2018 the adjustment indicators showed moderate levels, in 2025 they reach substantially higher values. These differences should be interpreted with caution, as pseudo-R^2^ measures in logistic regression are sensitive to changes in the distribution of the dependent variable. Given the substantial increase in overall access to digital health services between the two years (from 50.2% to 85.4%), some of the improvement in model fit could also reflect changes in the prevalence of outcomes.

In addition, it is important to acknowledge the inherent limitations of using self-reported data through surveys. Responses may be subject to various biases, such as recall bias or social desirability bias.

In both models, belonging to the group of people over 65 years of age is negatively associated with access to eHealth; however, in 2025 this effect not only remains, but increases considerably in magnitude. At the same time, in 2025, an intermediate age group—people between 35 and 49 years of age—becomes relevant, showing a significantly higher probability of access.

Likewise, there are significant changes in the role of territorial and health variables. While in 2018, residence in medium-sized municipalities and the presence of chronic diseases were positively associated with access to digital health, these variables are no longer significant in the 2025 model.

In this regard, variables related to the use of digital technologies are becoming increasingly important in explaining access to eHealth. Computer use remains a robust predictor in both periods, although its relative effect is reduced in 2025. Complementarily, the 2025 model incorporates the use of smartwatches, which emerges as a significant factor.

Overall, the comparison between the two models shows a change in the structure of the factors associated with access to eHealth between 2018 and 2025. While in 2018 access is related to a broader set of sociodemographic, territorial and health variables, in 2025 the model focuses on a smaller number of predictors with greater magnitude effects.

## 4. Discussion

The results of this study show a significant evolution in access to digital health services in Spain between 2018 and 2025, pointing to a progressive consolidation of eHealth as an organic infrastructure of the National Health System. However, beyond the increase in its reach—with 85.4% of the population accessing it through some digital channel—the most significant change lies in the transformation of the type and means of access. In this regard, there has been a shift from web portals, which were previously the predominant gateway, to mobile applications, which have now become the primary access points. This shift indicates a decentralisation of the healthcare system, which is consistent with the transition described by Meskó et al. [12]: from an institutional model of care to a distributed practice centred on citizens (with more autonomy managing their health, requesting medical appointments, accessing digital medical records or electronic prescriptions).

The Davis’ Models [53] are also relevant here, with mobile applications playing a decisive role in terms of perceived usefulness. The link between socio-demographic variables and cultural and attitudinal factors before technological changes is also highlighted, with regard to the devices and interfaces which, as Venkatesh et al. [54], point out, serve to understand the uneven adoption of digital health tools [55,56].

This empirical finding coincides with the shift in the “point of care” described by Mahou et al. [3], which positioned mHealth as the natural bridge between the healthcare system and the patient’s daily life, based on the democratisation that smartphones represent in terms of access points.

The expansion of access to eHealth, in conjunction with the significance attributed to age in the findings, serves to reinforce the notion of a “systemic shock” [15,16,17], taken up by Barral [10] to explain the forced digitalisation of the healthcare system. The advent of the pandemic provided the necessary impetus to overcome bureaucratic inertia, leading to the adoption of tools for both users and professionals, which played a key role, and whose digital skills and attitudes towards technology can either facilitate or hinder implementation [57].

Therefore, the expansion of digital health during this period was not the result of a gradual policy, but rather of a modernisation imposed by the emergency, which demonstrated the system’s capacity, but also highlighted the limitations of access and digital literacy among certain population groups.

The data show that, in 2025, access depends on a smaller set of explanatory factors, albeit with greater statistical weight. This apparent simplification reveals a reconfiguration of the determinants of access: while in 2018 the use of eHealth was conditioned by social, territorial and educational variables and by the type of device used, in 2025 it is mainly concentrated on age and the devices used. This finding coincides with the views of Norris [38], who warned that digitalisation does not eliminate gaps, but rather transforms them. In this case, inequality seems to shift from the availability of infrastructure to the differential capacity for appropriation and effective use of technology [31,34].

The persistence of the negative effect associated with the population over 65 years of age is one of the most relevant findings. Despite the general increase in access, ageing continues to act as a limiting factor; this pattern could suggest that the remaining barriers faced by older adults relate primarily to limited digital skills, rather than to technical shortcomings of the applications or web platforms. Although Großschädl et al. [33] document an increase in digital literacy among people over 50, driven by forced learning during the pandemic, this progress does not seem to have been sufficient to ensure full inclusion.

The convergence between these empirical results and theoretical postulates reinforces the idea that digital health is not only a technical innovation, but also a cultural mutation of the healthcare contract itself. In this sense, the ePatient profile proposed by Ferguson and Frydman [23] emerges here not as a potential figure, but as a statistical reality: most users manage their health from their mobile phones, which would allow them to access personalised information and participate more actively in decision-making. However, this growing horizontal flow of information also generates new tensions. As Lupton [24] warns, digital citizenship may also foster new forms of technological dependence, potentially shifting trust from healthcare professionals towards algorithmic systems. Recent research on the use of artificial intelligence in health-related decision-making and self-diagnosis suggests that, although these tools are increasingly used by patients, they often generate generic recommendations and require professional supervision, highlighting the risks associated with their uncritical adoption [25,49,58,59].

The rise of wearables and continuous monitoring systems confirms the expansion of Eysenbach’s paradigm [11] towards eHealth focused on prevention and personalisation. However, this process is not without ethical and regulatory dilemmas. The control of biometric data, privacy and algorithmic transparency pose challenges similar to those highlighted by Drezga-Kleiminger et al. [52]. In this sense, it would be reaffirmed that healthcare digitalisation, if not accompanied by inclusion policies, can reproduce the inequalities it seeks to correct. Thus, technological efficiency cannot be separated from social justice: a digitally advanced but cognitively exclusionary healthcare system would violate the principle of equity [10].

### Limitations

This study has several limitations that should be considered. Firstly, although the data used are representative of the Spanish population by age, gender and autonomous community, the sample size does not allow for stratified regional analyses. The diversity of regional health systems in Spain means that territorial differences in digital access may remain obscured. Future research should therefore examine regional contexts more explicitly.

Secondly, the quantitative and self-reported nature of the data restricts the ability to capture the experiences and meanings underlying digital exclusion. Survey responses may also be affected by recall or social desirability bias. Complementary qualitative approaches would provide a deeper understanding of these dynamics.

Finally, this research focused on sociodemographic factors and digital device use to examine their evolving influence on eHealth access between 2018 and 2025, with age emerging as a particularly important determinant. A limitation is the mixed-mode data collection in 2025 (CAWI for ages 18–64, CATI for 65+), which may introduce mode effects; however, weighting and consistent sampling quotas help mitigate potential distortions. Comparisons between waves should therefore be interpreted cautiously. Future research could also incorporate attitudinal factors, perceived benefits, and technology acceptance measures to provide a more comprehensive understanding of eHealth access.

## 5. Conclusions

Access to digital services has become widespread, reaching a majority of the population, corroborating the hypothesis of its expansion, but equity remains the main challenge. A significant number of people, especially the elderly, still lack access to eHealth, despite the technological advancements that have democratised the internet. The persistence of these inequalities demands a reinterpretation of founding principle of the 1986 “General Health Law”, that of “equal access for equal need”, in a contemporary key that could be updated as “equal access for equal digital capacity”.

In the current context, health equity depends not only on guaranteed coverage, but also on the real possibility of understanding, using and benefiting from the digital services available. From this perspective, digital literacy in health is revealed as a new social determinant of well-being, comparable in importance to education or income, as anticipated by Norman and Skinner [30].

In this sense, it is suggested that smartphones, in line with the hypothesis and objectives of this research, would have served to expand eHealth coverage, given the radical changes in access. However, the positive influence of devices such as computers, which continue to play an important role in both the pre-pandemic and post-pandemic models, as well as tablets (2018) and smartwatches (2025), could be pointing to a parallel phenomenon. In other words, although the role of smartphones has been and remains critical in this growing context of mHealth [3], reaching a large part of the population through apps no longer seems to have an effect per se, but has been diluted as it has become widespread. On the other hand, knowing how to use other types of devices that are either more complex or more specific revives the idea that not everything depends on access (although this is still not universal).

Beyond digital skills, one possible explanation lies in the design of the services themselves: in many cases, mobile applications do not allow advanced procedures to be completed and refer users to traditional web portals, which continue to have usability limitations on mobile interfaces. As a result, more complex procedures tend to require a change of platform to devices such as computers, where users with greater technological experience have a relative advantage. In addition, electronic identification systems in Spain have historically been geared towards use on computers, reinforcing this primacy. These interpretations, however, should be verified by qualitative evidence, as noted in the limitations of the study.

The findings of this study have particularly relevant implications for the design of public policies on digital health. Analysis of the results points to the urgent need for a redesign that should simultaneously address three dimensions: the technological, which ensures interoperability, transparency and data security; the social, focused on digital training and universal accessibility; and the cultural, focused on rebuilding the trust and empowerment of citizens/patients. Digital literacy, like reading literacy in its day, is now a necessary condition for the technological revolution to become a democratic revolution in the well.

Likewise, the results obtained underscore the relevance of developing lines of research that delve into the effective use and real exploitation of eHealth services, as well as the effects that the integration of equity can have on their design, delivery and evaluation. Understanding these processes will be essential to guide public policies capable of ensuring that digitalisation contributes to an inclusive healthcare system.

Finally, the comparison between the pre- and post-pandemic scenarios confirms that the COVID-19 pandemic has been a turning point in the digitalisation of the Spanish healthcare system. This episode has served as a historical laboratory that has accelerated, in a short period of time, transformations that under normal conditions would have taken decades. Forced digitalisation, the expansion of telemedicine, the incorporation of artificial intelligence and the emergence of a new digital patient profile are the cornerstones of a profound restructuring of the welfare state. In this context, the central question is no longer whether technology should be integrated into the healthcare system, but how to do so without compromising equity or public trust.

## Figures and Tables

**Table 1:** Recoded variables.

Variables	Recoded Categories
Gender	Man; Woman
Age by Groups	18–34; 35–49; 50–64; ≥65
Household composition	One person; Two people; Three people; Four people; Five people or more
Place of Residence	Less than 5000 inhabitants; Between 5000 and 25,000 inhabitants; Between 25,000 and 100,000 inhabitants; More than 100,000 inhabitants
Education	No education; Primary education; Lower secondary education; Upper secondary education; Higher education
Income	No Income; Less than €1200; Between €1200 and €1800; Between €1800 and €2500; Between €2500 and €4000; More than €4000
Employment Status	Employed; Unemployed; Student; Retired; Domestic Worker
Chronic Illness	Chronic Illness; No Chronic Illness
Health Status (self-perceived)	Very Good Health; Good Health; Regular Health; Poor Health; Very Poor Health
Device usage	Mobile Phone Usage; Tablet Usage; Computer Usage; Smartwatch Usage
Access to eHealth	Access_No; Access_Yes

**Table 2:** Sociodemographic characteristics of the sample in 2018 and 2025.

Variables	Categories	2018		2025	
		N	%	N	%
Gender	Man	828	48.8%	527	48.5%
	Woman	867	51.2%	559	51.5%
Age by Groups	18–34	354	20.9%	242	22.3%
	35–49	407	24.0%	305	28.1%
	50–64	505	29.8%	290	26.7%
	≥65	429	25.3%	248	22.9%

**Table 3:** Comparison of access to digital health in 2018 and 2025.

Access to...	2018				2025			
	YES		NO		YES		NO	
	N	%	N	%	N	%	N	%
Website	545	50.2%	540	49.8%	433	59.0%	301	41.0%
Mobile application	132	24.4%	409	75.6%	684	80.2%	169	19.8%
One or both	545	50.2%	540	49.8%	571	85.4%	98	14.6%

**Table 4:** Binary logistic regression model of access to eHealth in 2018.

Model Summary										
								Global Model Testing		
Model	Deviance	AIC	BIC	R^2^_McF_	R^2^_CS_	R^2^_N_	R^2^_T_	χ^2^	gl	*p*
1	1331	1347	1387	0.0861	0.112	0.15	0.114	125	7	<0.001
	Model Coefficients									
			95% CI						95% CI	
	Predictor	Estimator (B)	Lower	Upper	SE	Z	*p*	Odds Ratio	Lower	Upper
	Constant	−1.209	−16.293	−0.788	0.215	−5.63	<0.001	0.299	0.196	0.455
	Over 65 years old	−0.631	−11.356	−0.127	0.257	−2.45	0.014	0.532	0.321	0.881
	Between 25,000 and 100,000 inhabitants	0.403	0.0992	0.707	0.155	2.6	0.009	1.496	1.104	2.028
	Secondary Education	−0.759	−10,966	−0.422	0.172	−4.41	<0.001	0.468	0.334	0.656
	Chronic Illness	0.577	0.2902	0.865	0.147	3.94	<0.001	1.781	1.337	2.374
	Regular Health	−0.605	−10,008	−0.209	0.202	−3	0.003	0.546	0.368	0.811
	Tablet Use	0.394	0.1328	0.654	0.133	2.96	0.003	1.482	1.142	1.924
	Computer Use	1.216	0.8159	1.617	0.204	5.95	<0.001	3.374	2.261	5.036

Note. Models estimated using sample size of N = 1051. The estimators represent the log odds of ‘Access_Yes = 1’ vs. ‘Access_Yes = 0’.

**Table 5:** Binary logistic regression model of access to eHealth in 2025.

Model Summary										
								Global Model Testing		
Model	Deviance	AIC	BIC	R^2^_McF_	R^2^_CS_	R^2^_N_	R^2^_T_	χ^2^	gl	*p*
2	376	319	344	0.323	0.163	0.385	0.323	180	4	<0.001
	Model Coefficients									
			95% CI						95% CI	
	Predictor	Estimator (B)	Lower	Upper	SE	Z	*p*	Odds Ratio	Lower	Upper
	Constant	1.672	1.0279	2.32	0.329	5.09	<0.001	5.322	2.7951	10.133
	Between 35 and 49 years old	1.539	0.2037	2.87	0.681	2.26	0.024	4.661	1.2260	17.721
	Over 65 years old	−2.185	−2.7772	−1.59	0.302	−7.23	<0.001	0.112	0.0622	0.203
	Computer Use	1.015	0.4663	1.56	0.28	3.62	<0.001	2.760	1.5940	4.780
	Smartwatch Use	0.678	0.079	1.28	0.306	2.22	0.027	1.970	1.0822	3.585

Note. Models estimated using sample size of N = 1008. The estimators represent the log odds of ‘Access_Yes = 1’ vs. ‘Access_Yes = 0’.

**Table 6:** Summary of variables and odds ratios.

Variables	Model 1		Model 2	
	Estimator (B)	Odds Ratio	Estimator (B)	Odds Ratio
Between 35–49 years old	-	-	1.539	4.661
Over 65 years old	−0.631	0.532	−2.185	0.112
Between 25,000 and 100,000 inhabitants	0.403	1.496	-	-
Secondary Education	−0.759	0.468	-	-
Chronic Illness	0.577	1.781	-	-
Regular Health	−0.605	0.546	-	-
Tablet Use	0.394	1.482	-	-
Computer Use	1.216	3.374	1.015	2.760
Smartwatch Use	-	-	0.678	1.970

## Data Availability

The microdata generated by the projects responsible for the surveys will be deposited in the Centro de Investigaciones Sociológicas (CIS) database section (https://www.cis.es/es/estudios/arces/consultar-arces (accessed on 15 November 2025)), in accordance with the Initial R&D Survey Deposit Plan that governs the project’s funding calls. To this end, they may be consulted by indicating the name of the projects or their acronym. The data from the first survey (2018-acronym BDIGS) have already been deposited in the CIS. Although this repository is currently unavailable for consultation due to technical issues. The data from the first survey (2018) are available upon reasonable request from the principal investigators of the project at Prof. Ramón Bouzas (ramon.bouzas@usc.es). The data from the second survey (2025) are not publicly available at this stage because they form part of an ongoing research project and are subject to a temporary embargo to allow the completion and scientific exploitation of the project. The 2025 microdata (acronym “eSactiv”) will also be deposited in the CIS repository (https://www.cis.es/es/estudios/arces/consultar-arces (accessed on 15 November 2025)).

## Abbreviations

The following abbreviations are used in this manuscript:

B	Beta Estimator
CATI	Computer-Assisted Telephone Interviews
CAWI	Computer-Assisted Web Interviews
CIS	Centro de Investigaciones Sociológicas
ICT	Information and Communication Technologies
AI	Artificial Intelligence
mHealth	mobile health
SE	Standard Error
OR	Odds Ratio
VIF	Variance Inflation Factor

## Appendix A

### Appendix A.1

**Table A1:** Diagnosis of model 1.

Predictor	Omnibus Likelihood Ratio Test			Collinearity Statistics	
	χ^2^	gl	*p*	VIF	Tolerance
Over 65 years old	6.15	1	0.013	1.02	0.978
Between 25,000 and 100,000 inhabitants	6.83	1	0.009	1.01	0.994
Secondary Education	20.09	1	<0.001	1.02	0.977
Chronic Illness	15.88	1	<0.001	1.08	0.929
Regular Health	9.17	1	0.002	1.06	0.944
Tablet Use	8.76	1	0.003	1.03	0.973
Computer Use	39.35	1	<0.001	1.04	0.964

### Appendix A.2

**Table A2:** Model 2 diagnosis.

Predictor	Omnibus Likelihood Ratio Test			Collinearity Statistics	
	χ^2^	gl	*p*	VIF	Tolerance
Between 35 and 49 years old	7.15	1	0.008	1.10	0.911
Over 65 years old	60.40	1	<0.001	1.17	0.856
Computer Use	12.90	1	<0.001	1.09	0.917
Smartwatch Use	5.14	1	0.023	1.03	0.970

## References

[1] Esping-Andersen G., Myles J. (2018). The Welfare State and Redistribution. Social Stratification: Class, Race, and Gender in Sociological Perspective.

[2] Tsalampouni A. (2022). Health systems in the European Union and policy responses to Covid-19: A comparative analysis between Germany, Sweden, and Greece. J. Public Health Res..

[3] Mahou X., Barral B., Fernández Á., Bouzas-Lorenzo R., Cernadas A. (2021). eHealth and mHealth Development in Spain: Promise or Reality?. Int. J. Environ. Res. Public Health.

[4] García Armesto S., Abadía Taira B., Durán A., Hernández-Quevedo C., Bernal-Delgado E. (2011). España, Análisis Del Sistema Sanitario. Sistemas Sanitarios en Transición. https://sespas.es/2010/01/10/hit-espana-analisis-del-sistema-sanitario-2010/.

[5] Bernal-Delgado E., Garcia-Armesto S., Oliva J., Sanchez Martinez F.I., Repullo J.R., Pena-Longobardo L.M., Ridao-Lopez M., Hernandez-Quevedo C. (2018). Spain, Health System Review.

[6] Osorio A.P. (2024). Brecha digital en España: Análisis de las iniciativas estatales, autonómicas y locales para reducirla. RiiTE Rev. Interuniv. Investig. Tecnol. Educ..

[7] Borrell C., Peiró R., Ramón N., Pasarín M.I., Colomer C., Zafra E., Álvarez-Dardetc C. (2005). Desigualdades socioeconómicas y planes de salud en las comunidades autónomas del Estado español. Gac. Sanit..

[8] Moyano-Santiago M.A., Rivera-Lirio J.M. (2016). El enfoque de sostenibilidad en los planes de salud de las comunidades autónomas: El desarrollo sostenible como oportunidad. Gac. Sanit..

[9] Cantarero-Prieto D., Pascual-Sáez M., Gonzalez-Prieto N. (2017). Effect of having private health insurance on the use of health care services: The case of Spain. BMC Health Serv. Res..

[10] Barral Buceta B. (2021). Actores, Coalicións e Implementación Nas Políticas de eSaúde, Entre a Continuidade e a Innovación.

[11] Eysenbach G. (2001). What is e-health?. J. Med. Internet Res..

[12] Meskó B., Drobni Z., Bényei É., Gergely B., Győrffy Z. (2017). Digital health is a cultural transformation of traditional healthcare. mHealth.

[13] Silano M.F. (2013). La Salud 2.0 y la Atención de la Salud en la Era Digital. Rev. Médica Risaralda.

[14] Ramos A.C., Da Silva Á.F., Buceta B.B., Bouzas-Lorenzo R. (2023). Offerings and User Demands of eHealth Services in Spain: National Survey. J. Med. Internet Res..

[15] Sabatier P. (2019). Theories of the Policy Process.

[16] Sabatier P.A. (1998). The advocacy coalition framework: Revisions and relevance for Europe. J. Eur. Public Policy.

[17] Sabatier P.A. (1988). An advocacy coalition framework of policy change and the role of policy-oriented learning therein. Policy Sci..

[18] Sust P.P., Solans O., Fajardo J.C., Peralta M.M., Rodenas P., Gabaldà J., Eroles L.G., Comella A., Muñoz C.V., Ribes J.S. (2020). Turning the crisis into an opportunity: Digital health strategies deployed during the COVID-19 outbreak. JMIR Public Health Surveill..

[19] Amorim P., Brito D., Castelo-Branco M., Fàbrega C., da Costa F.G., Martins H., Gonçalves L., Gonçalves L.M., Martin V., Milner J. (2021). Telehealth Opportunities in the COVID-19 Pandemic Early Days: What Happened, Did Not Happen, Should Have Happened, and Must Happen in the Near Future?. Telemed. e-Health.

[20] European Commission for Communications Networks, Content and Technology, European Innovation Council (2025). 2025 Digital Decade eHealth Indicator Study—Final Report.

[21] Organización Panamericana de la Salud (2022). Informe Mundial Sobre el Edadismo.

[22] Etxebarria J.R. (2020). ¿No es país para viejos? La edad como criterio de triaje durante la pandemia de la COVID-19. Enrahonar Int. J. Theor. Pract. Reason.

[23] Ferguson T., Frydman G. (2004). The first generation of e-patients. BMJ.

[24] Lupton D. (2017). The digitised healthy citizen. Digital Health: Critical and Cross Disciplinary Perspectives.

[25] Prahl A., Jin K.T.W. (2024). Doctor Who? Norms, Care, and Autonomy in the Attitudes of Medical Students Towards AI Pre and Post ChatGPT. Hum.-Mach. Commun..

[26] McCausland D., Luus R., McCallion P., Murphy E., McCarron M. (2021). The impact of COVID-19 on the social inclusion of older adults with an intellectual disability during the first wave of the pandemic in Ireland. J. Intellect. Disabil. Res..

[27] Park S., Walker B., Anderson A., Shao Y., Callison K. (2023). Telemedicine Use by Age in Louisiana Medicaid During COVID-19: Claims-Based Longitudinal Analysis. J. Med. Internet Res..

[28] Yoon E., Hur S., Opsasnick L., Huang W., Batio S., Curtis L.M., Benavente J.Y., Lewis-Thames M.W., Liebovitz D.M., Wolf M.S. (2024). Disparities in Patient Portal Use Among Adults With Chronic Conditions. JAMA Netw. Open.

[29] Valla L.G., Rossi M., Gaia A., Guaita A., Rolandi E. (2025). The Impact of the COVID-19 Pandemic on Oldest-Old Social Capital and Health and the Role of Digital Inequalities: Longitudinal Cohort Study. J. Med. Internet Res..

[30] Norman C.D., Skinner H.A. (2006). eHEALS: The eHealth Literacy Scale. J. Med. Internet Res..

[31] Sen A. (2002). ¿Por qué la equidad en salud?. Rev. Panam. Salud Pública.

[32] Sen A. (2009). The Idea of Justice.

[33] Großschädl F., Marston H.R., Ivan L., Prabhu V., Earle S. (2025). Age as an important predictor for digital health literacy: Cross-sectional evidence of internet users from an international multisite study in North America and EU countries. Korean Soc. Educ. Gerontol..

[34] Haz-Gómez F.E., López-Martínez G., Manzanera-Román S. (2024). La exclusión digital como una forma de exclusión social: Una revisión crítica del concepto de brecha digital. Stud. Humanit. J..

[35] Wang T., Guo X., Wu T. (2021). Social Capital and Digital Divide: Implications for Mobile Health Policy in Developing Countries. J. Health Eng..

[36] Hall A.K., Bernhardt J.M., Dodd V., Vollrath M.W. (2014). The digital health divide: Evaluating online health information access and use among older adults. Health Educ. Behav..

[37] Andersen K.N., Nielsen J.A., Kim S. (2019). Use, cost, and digital divide in online public health care: Lessons from Denmark. Transform. Gov. People Process. Policy.

[38] Norris P. (2001). The Digital Divide: Civic Engagement, Information Poverty, and the Internet Worldwide.

[39] Bobrytska V.I., Krasylnykova H.V., Beseda N.A., Krasylnykov S.R., Skyrda T.S. (2024). Artificial intelligence (AI) in Ukrainian Higher Education: A Comprehensive Study of Stakeholder Attitudes, Expectations and Concerns. Int. J. Learn. Teach. Educ. Res..

[40] Pellas N. (2023). The influence of sociodemographic factors on students’ attitudes toward AI-generated video content creation. Smart Learn. Environ..

[41] Al Omari O., Alshammari M., Al Jabri W., Al Yahyaei A., Aljohani K.A., Sanad H.M., Al-Jubouri M.B., Bashayreh I., Fawaz M., Albashtawy M. (2024). Demographic factors, knowledge, attitude and perception and their association with nursing students’ intention to use artificial intelligence (AI): A multicentre survey across 10 Arab countries. BMC Med. Educ..

[42] Xu Y., Jiang T. (2025). The effects of anthropomorphic framing on senior news consumers’ attitudes towards health AI systems: A mediation of psychological distance. Inf. Res..

[43] Kaya F., Aydin F., Schepman A., Rodway P., Yetişensoy O., Kaya M.D. (2022). The Roles of Personality Traits, AI Anxiety, and Demographic Factors in Attitudes toward Artificial Intelligence. Int. J. Hum. Comput. Interact..

[44] Montag C., Nakov P., Ali R. (2025). On the need to develop nuanced measures assessing attitudes towards AI and AI literacy in repre-sentative large-scale samples. AI Soc..

[45] Encuentra E.H., Caballero J.L.G., Montagni I., Gutiérrez M.F., Sarmiento P.B. (2025). Digital health literacy among the Spanish population: A descriptive and latent class analysis study. Eur. J. Public Health.

[46] Babiker A., Alshakhsi S., Al-Thani D., Montag C., Ali R. (2024). Attitude Towards AI: Potential Influence of Conspiracy Belief, XAI Experience and Locus of Control. Int. J. Hum. Comput. Interact..

[47] Tahvanainen L., Tetri B., Ahonen O. (2024). Exploring and Extending Human-Centered Design to Develop AI-Enabled Wellbeing Technology in Healthcare.

[48] Huang G., Chen X., Liao C. (2025). AI-Driven Wearable Bioelectronics in Digital Healthcare. Biosensors.

[49] Zhang L., Yang J., Fang G. (2025). Factors influencing the acceptance of medical AI chat assistants among healthcare professionals and patients: A survey-based study in China. Front. Public Health.

[50] Cherrez-Ojeda I., Gallardo-Bastidas J.C., Robles-Velasco K., Osorio M.F., Leon E.M.V., Velastegui M.L., Pauletto P., Aguilar-Díaz F.C., Squassi A., Eras S.P.G. (2024). Understanding Health Care Students’ Perceptions, Beliefs, and Attitudes Toward AI-Powered Language Models: Cross-Sectional Study. JMIR Med. Educ..

[51] Altamimi I., Khan S.A., Alhemsi H., Alhumimidi A., Alsulaim K.B., Alomri F., Almutairi H., Alshankiti S., Alnobani O., Temsah M.-H. (2024). Exploring online health resources and self-care among irritable bowel syndrome patients: Analyzing internet use and AI chatbot interactions. mHealth.

[52] Drezga-Kleiminger M., Demaree-Cotton J., Koplin J., Savulescu J., Wilkinson D. (2023). Should AI allocate livers for transplant? Public attitudes and ethical considerations. BMC Med. Ethics.

[53] Davis F.D. (1989). Perceived Usefulness, Perceived Ease of Use, and User Acceptance of Information Technology. MIS Q..

[54] Venkatesh V., Morris M.G., Davis G.B., Davis F.D. (2003). User Acceptance of Information Technology: Toward a Unified View. MIS Q..

[55] Palas J.U., Sorwar G., Hoque R., Sivabalan A. (2022). Factors influencing the elderly’s adoption of mHealth: An empirical study using extended UTAUT2 model. BMC Med. Inform. Decis. Mak..

[56] Huang W., Ong W.C., Wong M.K.F., Ng E.Y.K., Koh T., Chandramouli C., Ng C.T., Hummel Y., Huang F., Lam C.S.P. (2024). Applying the UTAUT2 framework to patients’ attitudes toward healthcare task shifting with artificial intelligence. BMC Health Serv. Res..

[57] Ramos A.C., Buceta B.B., da Silva Á.F., Lorenzo R.B., Iturriria A.G. (2020). The Present and Future of eHealth in Spain From a Health Management Perspective. Int. J. Health Serv..

[58] Martin G.L., Létinier L. (2025). Artificial intelligence in personalized prescription: A narrative review of promise, peril, and practicality. Therapies.

[59] Campos V.M.S., Prudente T.P., Leão L.L., da Costa M.S., Oliva H.N.P., Monteiro-Junior R.S. (2025). Analyses of different prescriptions for health using artificial intelligence: A critical approach based on the international guidelines of health institutions. Health Inf. Sci. Syst..

